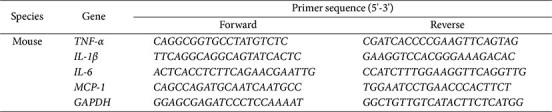# Effects of *Luffa cylindrica* (L.) Roem Extract on Microglial Activation-Mediated Mild Cognitive Impairment via Regulation of CREB Signaling Pathway

**DOI:** 10.4014/jmb.2506.06049

**Published:** 2025-09-26

**Authors:** Joon Park, Yongeun Kim, Jung-Eun Lee, Yun Tai Kim

**Affiliations:** 1 Department of Anesthesiology, University of Arizona, Tucson, AZ 85724, USA; 2 Food Functionality Research Division, Korea Food Research Institute, Wanju-gun, Jeollabuk-do 55365, Republic of Korea; 3 Department of Food Biotechnology, Korea University of Science and Technology, Daejeon 34113, Republic of Korea

**Keywords:** *Luffa cylindrica* (L.) Roem, neuroinflammation, cognitive impairment, microglial activation, natural products

## Abstract

Neuroinflammation is increasingly recognized as a pivotal contributor to mild cognitive impairment (MCI), with microglial activation playing a central role in this process. While *Luffa cylindrica* (L.) Roem is known for its anti-inflammatory properties, its effects on MCI and its active components have not been fully elucidated. In this study, we evaluated the anti-inflammatory and neuroprotective effects of *Luffa cylindrica* extract (LCE) on microglial activation and MCI-like behaviors induced by lipopolysaccharide (LPS). BV2 microglial cells were stimulated with LPS (1 μg/ml) and treated with LCE (25, 50, or 100 μg/ml). Microglial activation was assessed via Griess assay, western blotting, RT-PCR, and ELISA. *In vivo*, male ICR mice were received LCE (50 or 300 mg/kg) orally for 7 days in combination with intraperitoneal LPS (0.5 mg/kg). Cognitive function was evaluated using passive avoidance and Y-maze tests. The hippocampus was harvested for biochemical analysis. High-performance liquid chromatography (HPLC) was used to identify major bioactive components of LCE. LCE treatment significantly reduced the production of nitric oxide (NO), pro-inflammatory cytokine expression, and inflammation-associated protein levels in BV2 cells. These effects were associated with inhibition of the AKT-GSK3β-CREB signaling pathway. *In vivo*, oral LCE administration ameliorated LPS-induced cognitive impairment and decreased inflammatory markers in the hippocampus. HPLC analysis identified myricetin as a major component of LCE, which independently exhibited anti-inflammatory effects in microglia. These findings highlight the potential of LCE as a natural therapeutic agent for neuroinflammation-related cognitive impairment, with myricetin contributing to its pharmacological activity.

## Introduction

Mild cognitive impairment (MCI), which is characterized by a decline in memory, thinking, and other cognitive functions compared to normal cognitive aging, is also an underlying stage exhibited in the onset of irreversible neurodegenerative diseases [1]. Neuroinflammation, a key response of the brain’s immune system to injury or disease, has emerged as a critical factor in the onset of MCI. This complex process involves the activation of glial cells, particularly microglia and astrocytes, which release inflammatory mediators, cytokines, chemokines, and reactive oxygen species [2]. While neuroinflammation serves a protective role in the short term, chronic or excessive inflammation can disrupt neuronal function, synaptic plasticity, and neural circuitry, contributing to deficits in learning and memory, as well as executive functions [3, 4]. Increasing evidence links neuroinflammation to MCI in the initial stage of various neurological and neurodegenerative disorders, including Alzheimer’s disease (AD), Parkinson’s disease (PD), and multiple sclerosis. Aging is a significant factor associated with MCI and conditions like AD, and as the global population ages, the prevalence of cognitive impairment is expected to rise [5]. Therefore, there is a growing need to better understand and modulate neuroinflammation-related MCI for potential therapeutic interventions.

Microglial activation is a central driver of neuroinflammation, as these resident immune cells of the central nervous system respond rapidly to injury, infection, or cellular stress to survey the brain environment [6]. Upon detecting damage-associated molecular patterns (DAMPs) or pathogen-associated molecular patterns (PAMPs), such as lipopolysaccharide (LPS) and amyloid β (Aβ), microglia become activated and release a variety of pro-inflammatory mediators. These include excessive nitric oxide (NO), inducible nitric oxide synthase (iNOS), cyclooxygenase-2 (COX-2), tumor necrosis factor alpha (TNF-α), interleukin-1 beta (IL-1β), IL-6, and monocyte chemoattractant protein-1 (MCP-1), which initiate and sustain an inflammatory cascade within the brain [7, 8]. This sustained inflammatory response contributes to the progression of neurodegenerative diseases, such as AD and PD, in which chronic microglial activation is a hallmark feature associated with cognitive and motor deficits [6]. Thus, understanding the mechanisms regulating microglial activation is crucial for developing strategies to mitigate neuroinflammation-related neurodegeneration and MCI.

Ionized calcium-binding adaptor molecule 1 (IBA-1) is a cytoplasmic protein specifically expressed in microglia, the resident immune cells of the central nervous system [9]. IBA-1 is a widely used marker to detect microglial presence and activation; its expression significantly increases in response to neuroinflammatory stimuli, including models of neurodegenerative disease and injury [10, 11]. This increase accompanies morphological changes in microglia from a divided to an amoeboid shape, indicating their transition from a surveillant to an activated state involved in inflammatory and phagocytic responses [12]. In experimental settings such as LPS (lipopolysaccharide)-induced cognitive impairment models, IBA-1 immunostaining is used to evaluate microglial activation, distribution, and density, which are sensitive indicators of neuroinflammation and underlying pathological changes [11, 12].

*Luffa cylindrica* (L.) Roem, commonly known as sponge gourd or loofah, is an annual species of vine cultivated widely in Asia, India, Brazil, and the USA [13]. Beyond its nutritional value, *Luffa cylindrica* has long been used in traditional medicine to treat inflammatory and metabolic disorders. Previous studies have revealed that *Luffa cylindrica* is rich in bioactive compounds such as myricetin, and exhibits potent antioxidant and anti-inflammatory effects, making it a promising candidate for managing inflammation-related disorders [13-15]. While the anti-inflammatory effects of *Luffa cylindrica* extract (LCE) has been documented, its impact on neuroinflammation-induced MCI and the underlying mechanisms remain unexplored. Given that microglial activation is a key driver of neuroinflammation in MCI, investigating the potential of LCE to suppress microglial activation and improve cognitive function could provide valuable insights into its therapeutic utility.

In this study, we evaluated the effects of LCE on microglial activation. *In vitro*, LCE was assessed in LPS-treated BV2 microglial cells to measure the suppressive effects on inflammatory mediators and the modulation of the AKT-GSK3β-CREB signaling pathway. We used LPS injections to induce MCI-like behaviors in mice, and the effects of LCE on hippocampal inflammatory mediators were then assessed. Additionally, HPLC analysis was utilized to identify a major component of LCE, and its anti-inflammatory effects in LPS-treated BV2 microglial cells were also investigated.

## Materials and Methods

All chemical reagents, including lipopolysaccharide (LPS), cAMP response element-binding protein (CREB) inhibitor (Millipore, 538341), imipramine (Sigma, I7379), and myricetin (Sigma, 70050) were obtained from Sigma-Aldrich (USA). BV2 microglial cells were donated by Jin Young Hur, PhD, from the Korea Food Research Institute (Republic of Korea). RPMI 1640 and penicillin-streptomycin were sourced from Thermo Scientific Hyclone (USA), and fetal bovine serum (FBS) from Gendepot (Barker, USA). Antibodies against iNOS (CST, 13120), COX-2 (CST, 12282), p-GSK3β (Ser9) (CST, 5558), GSK3β (CST, 12456), p-CREB (Ser133) (CST, 9198), CREB (CST, 9197), p-AKT (Ser473) (CST, 9271), and AKT (CST, 9272) were purchased from Cell Signaling Technology (USA), while antibodies for IBA1 (Abcam. Ab178846) and β-actin (Santa Cruz, sc-47778) were sourced from Abcam (UK) and Santa Cruz Biotechnology (USA), respectively.

### Sample Preparation

Dried *Luffa cylindrica* fruits were purchased from Chowon Herb (Republic of Korea). The fruits were cultured in the Chulweon area of Gangwon Province, and harvested and dried in late August. To prepare *Luffa cylindrica* extract (LCE), whole fruits were immersed in 70% ethanol at a 1:10 g/ml ratio and maintained at 80°C for 6 h with constant shaking. The solution was filtered through Whatman filter paper No. 1, and the filtrate was evaporated and freeze-dried to yield a dry extract, calculated to be 9.03%. The dried LCE was dissolved in DMSO for cell treatments. The samples are stored at the Korea Food Research Institute (voucher no. KFRI-MAT-0118).

### Cell Culture and Viability Assay

BV2 cells were cultured in RPMI 1640 supplemented with 5% FBS and 1% penicillin-streptomycin at 37°C in a 5% CO_2_ incubator. Cell viability was assessed by seeding BV2 cells (5 × 10^4^ cells/well) in 96-well plates overnight, then treating them with the samples indicated in the figures for 24 h. The MTS assay reagent (Promega, USA) was added to each well, and absorbance at 490 and 690 nm was measured using a microplate reader (Bio-Rad Inc., USA).

### Griess Assay

The Griess assay was conducted following the manufacturer’s instructions. Briefly, BV2 cells were seeded in 96-well plates at a density of 5 × 10^4^ cells/well. Following 24 h, each sample was pre-treated at specified concentrations for 1 h, followed by co-treatment with LPS (1 μg/ml). The cell concentrations were determined based on previous studies [16, 17], and the nitric oxide (NO) production levels were measured 24 h post-treatment.

### Western Blotting

To perform western blot analysis, BV2 cells were seeded in 6-well culture plates at a density of 4 × 10^5^ cells/well and treated with samples and/or LPS. After treatment, cells were harvested to extract proteins, and western blotting was performed with each target antibody, following procedures described in a previous study [18].

### Total RNA Isolation and Quantitative Real-Time (qRT-PCR) Assay

RNA was extracted using Trizol reagent, and cDNA synthesis was performed using ReverTra Ace^®^ qPCR RT Master mix (Toyobo, Japan) according to the manufacturer's instructions. The QuantStudio 6 Flex Real-Time PCR system (Applied Biosystems, USA) was used to measure gene expression levels by qRT-PCR using POWER SYBR Green PCR Master Mix (Applied Biosystems) and specific primers (Table 1). Gene expression changes were calculated using the 2^-ΔΔCT^ method and are presented as fold-changes relative to control samples.

### Determination of Pro-Inflammatory Cytokine Levels

Pro-inflammatory cytokine levels released by activated microglia were measured using ELISA following the manufacturer’s protocols (R&D Systems, Inc., USA). Conditioned media were collected from cells treated with LCE and/or LPS for 24 h, and then stored at -80°C until analysis.

### Animal

All animal procedures were conducted in accordance with the Korea Food Research Institute’s Guidelines for Animal Care and Use, and the study protocol (KFRI-M-19042) was approved by the relevant ethics committee (clinical trial number not applicable). Male ICR mice (6 weeks old, weighing 18–22 g) were housed in a temperature-controlled room (23 ± 2°C) with a 12-h light/dark cycle and were provided food and water ad libitum. Mice were acclimated to laboratory conditions for at least one week before sample administration.

### Experimental Design and Sample Administration

LCE was dissolved in distilled water for oral administration. Sham and control groups received an equivalent volume of vehicles. To determine the relevant dose of LCE in animals, we selected the dose for oral administration according to other studies [19, 20]. Mice (*n* = 5 per group, 5 groups in total) were randomly assigned to the following groups: (i) control group (Sham), (ii) LPS-treated group (LPS), (iii) LPS with low-dose LCE (50 mg/kg/day), (iv) LPS with high-dose LCE (300 mg/kg/day), and (v) LPS with imipramine (30 mg/kg/day) as a positive control. LPS was dissolved at 0.5 mg/kg in 0.9% (w/v) saline and injected intraperitoneally to induce inflammation-related MCI. After seven days of daily treatment, behavioral assessments were conducted, and hippocampal tissue was collected from mice anesthetized with 2% isoflurane to examine underlying mechanisms.

### Passive Avoidance Test (PAT)

The PAT was conducted as previously described to assess inflammation-induced cognitive impairment [21]. In the training trial, mice were placed in a safe compartment, and the door to a dark compartment was opened. Upon entry into the dark compartment, the door was closed, and a mild electric shock (0.5 mA for 3 s) was administered to the mice’s feet. The following day, mice were placed back in the safe compartment, and the latent time to enter the dark compartment was recorded.

### Y-Maze Test

The Y-maze test, commonly used to assess short-term memory in mice, was conducted following established protocols [22]. Mice were placed in the center of a Y-shaped maze, elevated 50 cm above the ground, and allowed to explore freely for 10 min. The number of entries into each arm and spontaneous alternations were recorded by an observer.

### HPLC-UV-Based Analysis

LCE and myricetin were dissolved in absolute methanol to prepare stock solutions. Myricetin analysis was conducted according to a previous protocol with minor modifications [18]. The mobile phase consisted of a mixture of (A) 0.1% formic acid in distilled water and (B) ACN. The gradient conditions were as follows: 85%-85%A for 0-10 min, 85%-75% A for 10-20 min, and 75%-65% A for 20-30 min. The flow rate was set to 1 ml/min with a column temperature of 30°C, and UV detection was performed at 350 nm.

### Statistical Analysis

Data are represented as mean ± SEM. Statistical analyses were performed using one-way analysis of variance (ANOVA) followed by Dunnett's post hoc test with Prism 5 (GraphPad Software Inc., USA). Significance was set at *p* < 0.05. Hash symbols (#, ##, and ###) denote significance (*p* < 0.05, *p* < 0.01, and *p* < 0.001, respectively) between the non-treated and LPS-treated groups. Asterisks (*, **, and ***) indicate significance (*p* < 0.05, *p* < 0.01, and *p* < 0.001, respectively) between groups co-treated with LPS and LCE and the LPS-only group.

## Results

### LCE Treatment Inhibits Microglial Activation in LPS-Treated BV2 Cells

**Fig. 1:**
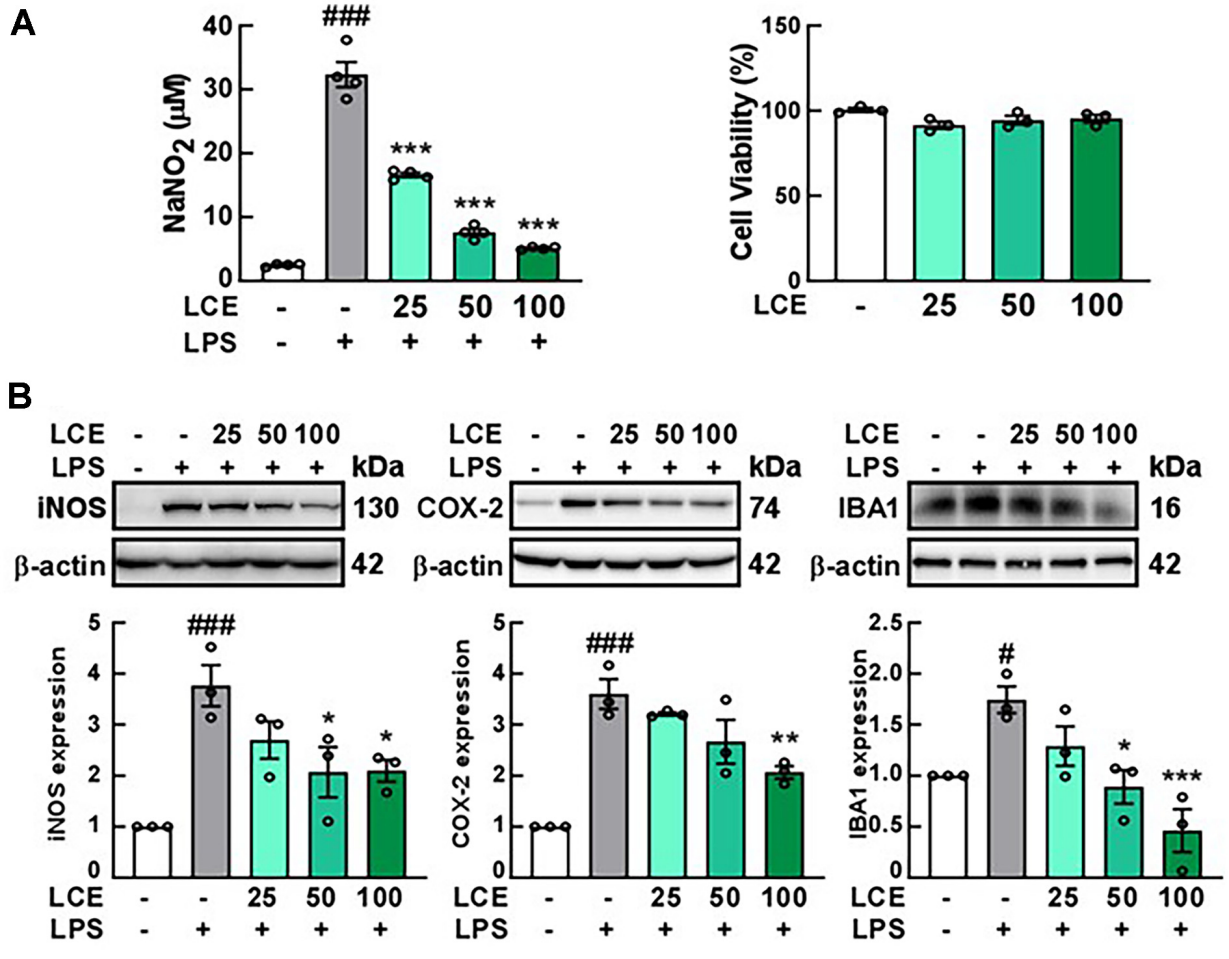
Effects of *Luffa cylindrica* extract (LCE) treatment on LPS-induced inflammatory mediators in BV2 microglial cells. BV2 cells were pre-treated with LCE at concentrations indicated in the Figure (μg/ml) for 1 h, followed by treatment with 1 μg/ml LPS to induce inflammatory responses for 24 h. (**A**) Effect of LCE on LPS-induced nitric oxide (NO) production in BV2 cells. NO production and cell viability were measured using the Griess and MTS assays, respectively. (**B**) Effect of LCE on the expression of inducible nitric oxide synthase (iNOS), cyclooxygenase-2 (COX-2), and ionized calciumbinding adaptor molecule 1 (IBA1) in LPS-treated BV2 cells. Protein levels were analyzed by western blotting using specific antibodies for each protein.

We first examined the effect of LCE on microglial activation in BV2 cells treated with LPS, focusing on whether LCE could regulate LPS-induced NO production. Our results indicated that LCE treatment significantly decreased NO production in a dose-dependent manner without exhibiting cytotoxicity (Fig. 1A). LCE concentrations up to 100 μg/ml were non-toxic but effectively suppressed NO production; hence, these concentrations were selected for subsequent experiments. We then assessed the effect of LCE on protein expressions associated with inflammation in BV2 cells, finding that LCE reduced LPS-induced iNOS and COX-2 levels in a dose-dependent manner (Fig. 1B). Additionally, LCE treatment decreased IBA1 expression, a recognized marker of microglial activation, in BV2 cells.

**Fig. 2:**
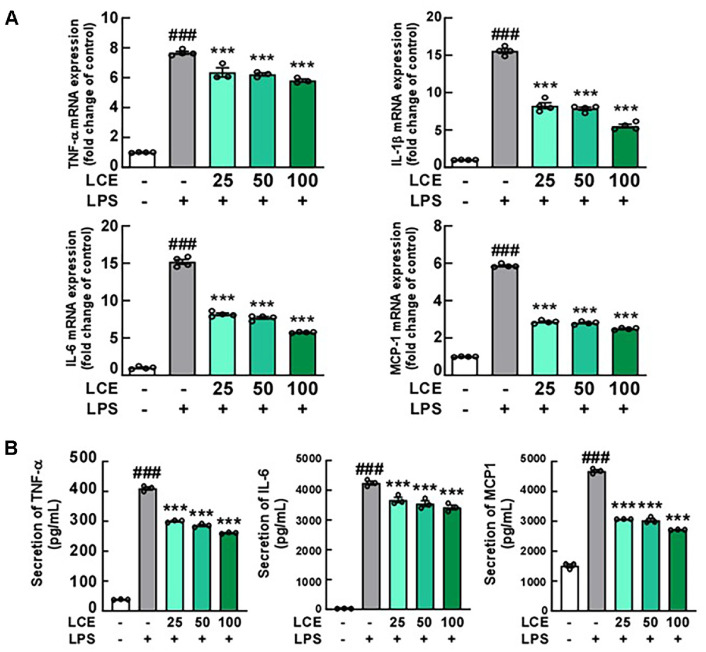
Effects of *Luffa cylindrica* extract (LCE) treatment on LPS-induced inflammatory cytokines in BV2 microglial cells. BV2 cells were pre-treated with LCE at concentrations indicated in the Figure (μg/ml) for 1 h, followed by treatment with 1 μg/ml LPS to induce expressions of tumor necrosis factor alpha (TNF-α), interleukin-1 beta (IL-1β), IL-6, and monocyte chemoattractant protein-1 (MCP-1) for 24 h. (**A**) Effect of LCE on mRNA levels of inflammatory cytokines in BV2 cells treated with LPS. mRNA levels were evaluated using qRT-PCR. (**B**) Effect of LCE on protein levels of inflammatory cytokines in LPS-treated BV2 cells. The cultured media were collected and the ELISA assay was utilized to measure protein levels of inflammatory cytokines in BV2 cells.

Furthermore, we measured the production of pro-inflammatory cytokines using RT-PCR and ELISA, observing that LCE treatment significantly reduced elevated levels of pro-inflammatory cytokines such as TNF-α, IL-1β, IL-6, and MCP-1 in LPS-activated BV2 cells (Fig. 2A and 2B). These findings demonstrate that LCE treatment effectively regulates microglial activation in LPS-stimulated BV2 cells.

### LCE Treatment Modulates CREB Signaling Pathway Activation in BV2 Microglial Cells

**Fig. 3:**
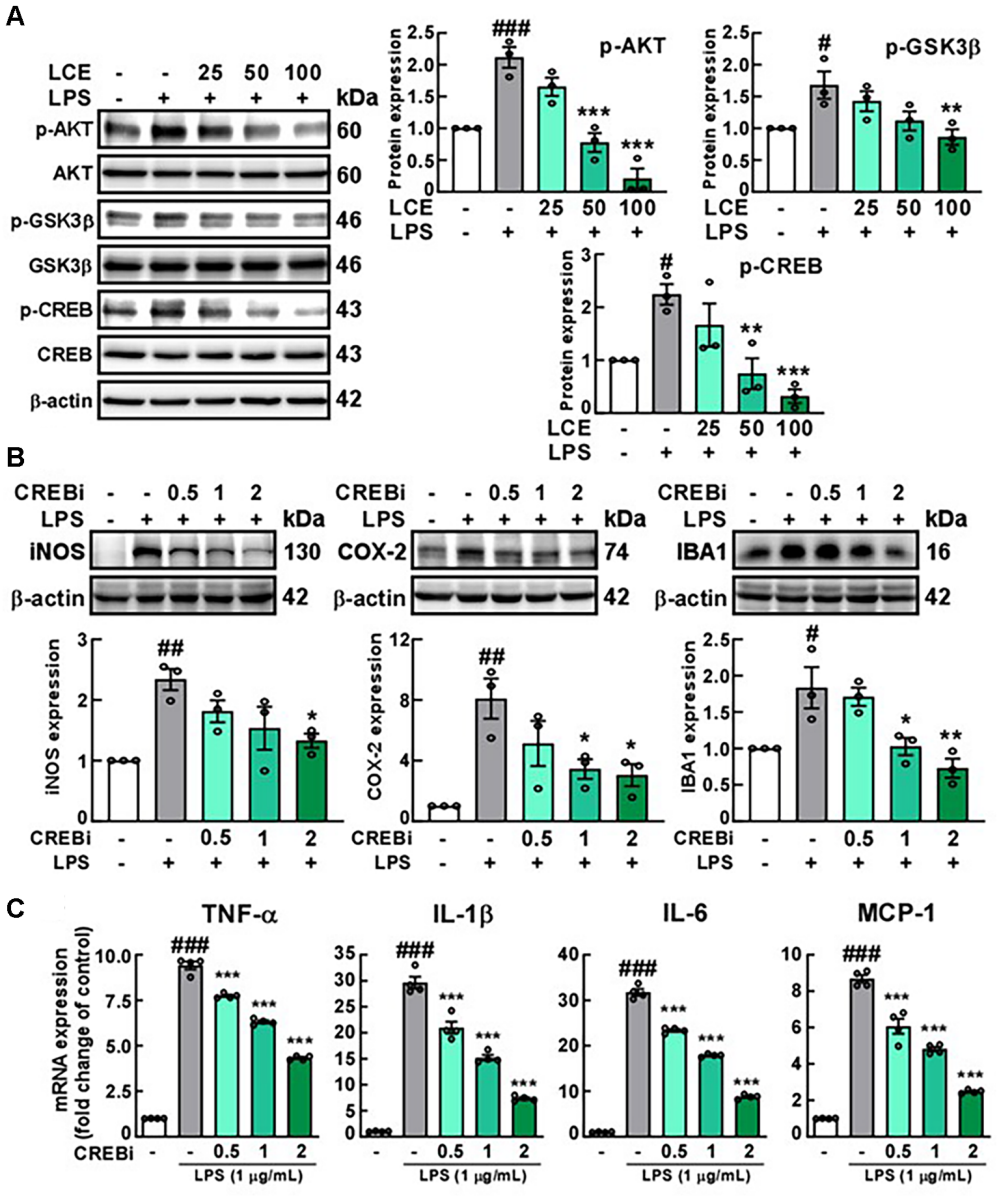
Effects of *Luffa cylindrica* extract (LCE) treatment on LPS-induced AKT-GSK3β-CREB signaling in BV2 microglial cells. BV2 cells were pre-treated with LCE at concentrations indicated in the Figure (μg/ml) for 1 h, followed by treatment with 1 μg/ml LPS to induce the activation of the underlying signaling. (**A**) Effect of LCE on the activation of AKTGSK3β- CREB signaling in BV2 cells treated with LPS for 0.5 h. Phosphorylated protein expressions were evaluated using western blotting with each antibodies. (**B**) Effect of CREB inhibitor on the expression of inflammatory mediators in LPStreated BV2 cells. (**C**) Effect of CREB inhibitor on mRNA expression of inflammatory cytokines in LPS-treated BV2 cells. To identify the effect of CREB inhibitor, BV2 cells were pre-treated with CREB inhibitor at concentrations indicated in the Figure (μM) for 1 h, followed by treatment with 1 μg/ml LPS for 24 h.

To investigate the mechanisms underlying the inhibitory effect of LCE on microglial activation, we examined whether LCE treatment regulates phosphorylated CREB in BV2 cells. CREB is a transcription factor critical for regulating neuroinflammation in response to stimuli [16, 23-25]. The results showed that LPS treatment led to an increase in phosphorylated CREB. Meanwhile, phosphorylation of GSK3β and AKT, which are upstream kinases in the CREB pathway, was also upregulated by treatment with LPS in BV2 cells. However, these LPS-induced upregulations were dose-dependently decreased by LCE treatment (Fig. 3A). Most studies have demonstrated that microglial activation can be inhibited by activating CREB [26-28], which is inconsistent with the present findings. This prompted us to perform further tests to investigate the effect of inhibition of CREB on LPS-induced microglial activation. To confirm the role of CREB inhibition on microglial activation, LPS-treated BV2 cells were pre-treated with a CREB inhibitor described to diminish CREB phosphorylation. The results showed that microglial activation was significantly suppressed by the CREB inhibitor in LPS-stimulated BV2 cells, as evidenced by a dose-dependent reduction in expressions of inflammation-related proteins (Fig. 3B and 3C). These findings suggest that LCE treatment inhibits microglial activation by modulating the CREB signaling pathway.

### Oral Administration of LCE Mitigates MCI-Like Behaviors and Reduces Microglial Activation in LPS-Injected Mice

**Fig. 4:**
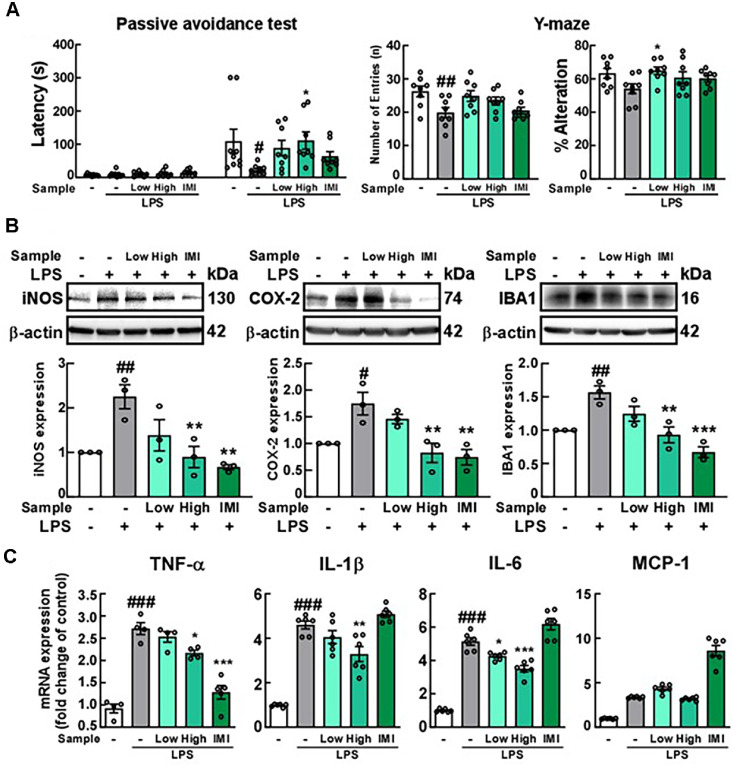
Effects of *Luffa cylindrica* extract (LCE) on cognitive impairment and microglial activation in LPStreat mice. (**A**) Effect of LCE administration on LPS-induced congnitive dysfunction in mice. Each sample was preadministered oral for 7 days and then LPS (0.5 mg/kg) was injected intraperitoneally with oral administration of each sample for an additional 7 days [Low group: 100 mg/kg LCE, High group: 300 mg/kg LCE, IMI group: 30 mg/kg]. Vehicles were administered to each group at the same volume as the sample. (**B** and **C**) Effect of LCE on LPS-induced microglial activation in the hippocampus of mice. Protein and mRNA expressions were determined with the hippocampus by western blot and qRTPCR, respectively.

The effects of LCE on LPS-induced MCI were evaluated in mice using the PAT and Y-maze tests. Increased levels of LPS have been observed in both serum and cerebrospinal fluid of MCI and AD patients [29]. In the present study, we utilized LPS treatment to induce MCI-like behaviors in mice. Moreover, the PAT is designed to measure long-term memory in mice, *i.e.*, their ability to avoid a negative stimulus, in this case a mild electric shock to the foot. The Y-maze test is a behavioral tool that evaluates spatial working memory using the instinctive tendency of mice to explore new environments. Imipramine was reported to attenuate MCI-like behaviors in LPS-treated mice. Thus, imipramine was utilized as a positive control in the present study [21]. The PAT results indicated that the LPS-induced decrease in escape latency was increased by LCE treatment. Additionally, the number of entries and alterations recovered in mice administered with LCE in the Y-maze test (Fig. 4A). LCE administration also restored short-term spatial memory in the Y-maze, with a significant effect only at the low dose. Imipramine treatment resulted in a non-significant attenuation in behavior tests. Following that, we investigated the underlying mechanisms by examining the hippocampus, which is the most studied part of the brain as it supports many cognitive functions, including memory, learning, and spatial navigation. We found that expression of iNOS, COX-2, and IBA1 was reduced by oral administration of LCE and imipramine in LPS-treated mice (Fig. 4B). Additionally, LCE administration significantly reduced levels of pro-inflammatory cytokines in the hippocampus, except for MCP-1 (Fig. 4C). These findings indicate that LCE can alleviate inflammation-induced MCI by suppressing microglial activation in the hippocampus.

### A Major Component of LCE and Its Anti-Inflammatory Effect on Microglial Activation

**Fig. 5:**
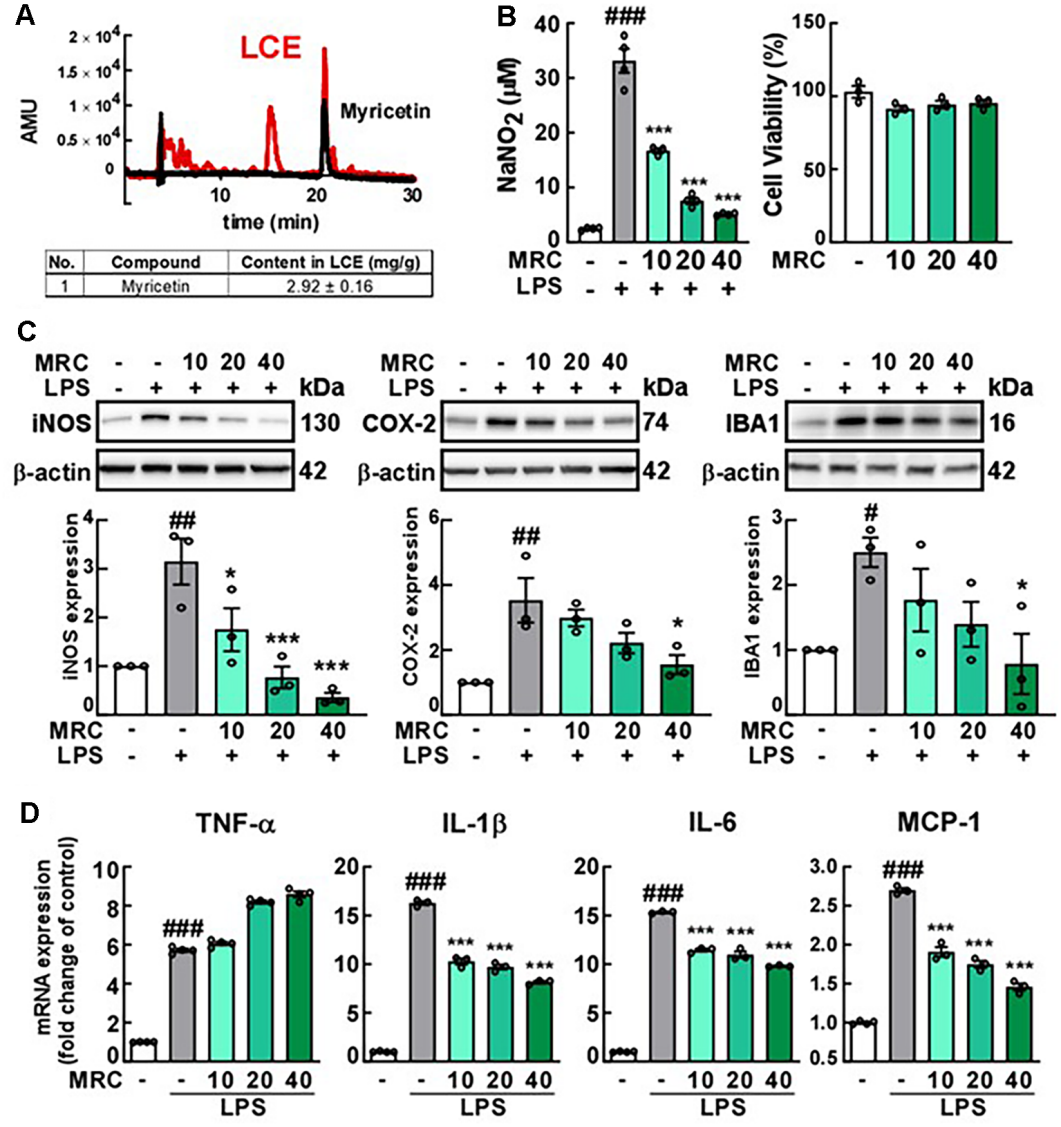
The identification of a major component of *Luffa cylindrica* extract (LCE) and its anti-inflammatory effects. (**A**) Qualification and quantification of a mojor component of LCE. An HPLC method was utilized to identify a major component of LCE. To assess the anti-inflammatory effects of myricetin (MRC), BV2 cells were pre-treated with MRC at concentrations indicated in the Figure (μM) for 1 h, followed by treatment with LPS (1 ug/ml) for 24 h. (**B**) Effect of MRC on the production of nitric oxide (NO) induced by LPS treatment and cell viability in BV2 cells. NO production and cell viability were measured using the Griess and MTS assays, respectively. (**C**) Effect of MRC on the expression of inducible nitric oxide synthase (iNOS), cyclooxygenase-2 (COX-2), and ionized calcium-binding adaptor molecule 1 (IBA1) in LPS-treated BV2 cells. Protein levels were analyzed by western blotting using specific antibodies for each protein. (**D**) Effect of MRC on mRNA expression of inflammatory cytokines in LPS-treated BV2 cells, as determined using qRT-PCR and described in Materials and Methods.

To identify the major components of LCE, HPLC analysis was performed and revealed myricetin as a primary component, accounting for 2.92 ± 0.16 mg/g of LCE (Fig. 5A). Treatment of BV2 cells with myricetin reduced NO production and the expression of inflammatory markers iNOS, COX-2, and IBA1 in LPS-stimulated cells without cell toxicity (Fig. 5B and 5C). Furthermore, myricetin treatment led to a reduction in pro-inflammatory cytokine levels, except for TNF-α (Fig. 5D). These results suggest that myricetin may be one of the major active compounds in LCE and contributes to its anti-inflammatory effects.

## Discussion

In this study, we found that LCE treatment significantly reduced microglial activation in LPS-activated BV2 microglial cells, as evidenced by a dose-dependent decrease in iNOS, COX-2, IBA1, and pro-inflammatory cytokines. Additionally, our results suggest that the inhibitory effects of LCE were mediated through the suppression of LPS-induced phosphorylation cascades in the AKT-GSK3β-CREB signaling pathway. This was confirmed by use of the CREB inhibitor to block CREB phosphorylation, which led to a reduction in LPS-induced iNOS, COX-2, IBA1, and pro-inflammatory cytokines in BV2 cells. *In vivo*, oral administration of LCE improved MCI and reduced markers of microglial activation in the hippocampus of LPS-injected mice. Furthermore, myricetin, identified as a major component of LCE, demonstrated significant anti-inflammatory effects on microglial activation in BV2 cells.

LCE treatment effectively suppressed LPS-induced iNOS and COX-2 expression in BV2 cells and the hippocampus. iNOS, produced by activated microglia in response to pro-inflammatory stimuli, contributes to oxidative damage through toxic peroxynitrite and ROS generation in the nervous system [30]. Kummer *et al*., reported that iNOS-mediated NO production accelerates Aβ aggregation by nitrating tyrosine 10, leading to cognitive impairments in APP/PS1 mice [31]. In alignment with this, oral administration of an iNOS inhibitor, or the use of iNOS knockout mice, has been shown to reduce cognitive dysfunction [31]. COX-2, another pro-inflammatory enzyme, is linked to neurodegenerative diseases and cognitive decline through its role in initiating pro-inflammatory processes and neuronal degeneration [32]. Specifically in microglia, COX-2 inhibition has been associated with restored microglial and memory functions impaired by Aβ [33, 34]. In LPS-injected mice, cognitive impairments were significantly alleviated by inhibiting iNOS and COX-2 [35]. Thus, suppressing iNOS and COX-2 in microglia in the hippocampus may contribute to reducing microglial activation and MCI.

LCE treatment also significantly decreased pro-inflammatory cytokine levels in microglia and the hippocampus following LPS stimulation. Activated microglia are a primary source of pro-inflammatory cytokines, which are linked to cognitive impairments. Clinically, patients with cognitive disorders often exhibit elevated serum levels of pro-inflammatory cytokines compared to healthy controls [36]. Treatment with a microglia inhibitor has been shown to decrease IL-1β and IL-6 serum levels and improve cognitive deficits [36]. Animal studies have corroborated this; TNF knockout mice exhibited reduced LPS-induced cognitive deficits compared to wild-type controls [37]. Blocking IL-1β reversed hippocampal LTP impairment and improved cognition in AD mice [38]. In addition, IL-6 inhibition ameliorated cognitive impairment in systemic inflammation models [39]. MCP-1 knockout mice injected with LPS showed reduced TNF-α and IL-1β levels, reflecting decreased inflammation [40]. Treatment with a microglia inhibitor resulted in a reduction of the expression of TNF-α and IL-1β and protection of neurogenesis in the hippocampus of AD mice [41]. Moreover, neuronal damage and expressions of iNOS, TNF-α, and IL-1β were significantly reduced by suppressing microglial activation in the hippocampus of PD mice. These effects recovered cognitive deficit-like behaviors in both AD and PD models [42]. Collectively, these findings underscore the therapeutic potential of inhibiting microglial activation to improve outcomes.

Our study further revealed that LCE treatment suppresses CREB signaling, resulting in the reduction of microglial activation in LPS-treated BV2 cells. Furthermore, the role of CREB suppression was identified through the CREB inhibitor by showing a significant decrease in expressions of inflammatory proteins in LPS-treated BV2 cells. We measured whether LCE treatment modulates the other representative signaling pathways related to inflammation, NF-κB, and MAPK; however, none of these pathways were regulated by LCE treatment in LPS-treated BV2 cells (data not shown). Although the evidence suggested that CREB activation can suppress microglial activation [26-28], the role of CREB in the brain remains controversial and complex [43]. In macrophages which have a similar role, CREB functions as a key inducer of inflammation by showing that CREB suppression reduced the upregulation of COX-2, TNF-α, IL-1β, and IL-6 induced by LPS [44, 45]. Our results aligned with these findings, as CREB inhibition decreased inflammatory markers, including iNOS, COX-2, IBA1, and pro-inflammatory cytokines. In addition, Manners *et al*. conducted a study using a mouse model in which CREB was specifically deleted from the hippocampus to determine the role of CREB [46]. The hippocampal CREB deletion led to the downregulation of gene clusters associated with inflammation and the immune system [46]. Moreover, intracerebroventricular injections of Aβ significantly induced cognitive impairment and the phosphorylation of CREB in microglia at 24 h in the hippocampal non-granular cell layers and non-pyramidal layer, which was reversed by treatment with minocycline and inhibition of CREB, respectively [47]. The response of CREB was different depending on cell types, meaning that CREB has diverse roles in the CNS [47]. Our study also demonstrated that suppression of CREB significantly reduced Aβ-induced IL-6 expression in BV2 cells, as well as the damages to neuronal development in the primary hippocampal neuron by Aβ-treated BV2 culture media [47]. Collectively, the research indicates that the effects of CREB activation vary depending on the situation. Therefore, further studies are still needed to elucidate the role of CREB in microglia on neuroinflammation.

Myricetin was identified to be one of the major components of LCE and exhibited the inhibitory effect on microglial activation in LPS-treated BV2 microglial cells. Consistent with our findings, Jang *et al*. reported anti-inflammatory effects of myricetin on microglial activation, as shown by suppressions of production of NO gases, iNOS, COX-2, TNF-α, and IL-1β in LPS-treated BV2 cells [48]. Furthermore, the administration of myricetin was found to remarkably inhibit microglial activation in the hippocampus and cortex, as evidenced by the suppression of IBA1 expression [48]. These observations indicate that myricetin’s anti-inflammatory effects on microglia align with those seen with LCE treatment. According to a study on drug transport across the BBB, it suggests that small molecules with a molecular weight <400 g/mol, and forming fewer than 8 hydrogen bonds, are more likely to cross the BBB via lipid-mediated free diffusion [49]. Myricetin fulfills these criteria, as it has a molecular weight of 318.237 g/mol and forms fewer than 6 hydrogen bonds. Based on these physicochemical properties, it is reasonable to infer that myricetin can penetrate the BBB and exert neuroprotective effects in the CNS. Therefore, myricetin is likely a major bioactive compound in LCE.

## Conclusion

In conclusion, LCE treatment suppresses microglial activation in LPS-stimulated BV2 cells by reducing inflammatory mediators and cytokines. The inhibitory effect of LCE was identified to result from the suppression of AKT-GSK3β-CREB signaling pathway in LPS-treated BV2 microglial cells. *In vivo*, oral LCE administration improved MCI-like behaviors in LPS-injected mice and decreased hippocampal inflammatory markers. Myricetin, identified as a primary component of LCE, also exhibited anti-inflammatory effects in LPS-stimulated BV2 cells. Whether LCE treatment regulates CREB activation in microglia in the hippocampus, and whether inhibition of CREB modulates microglial activation, still need to be identified. Nevertheless, these findings suggest the therapeutic potential of LCE for MCI management.

## Supplemental Materials

Supplementary data for this paper are available on-line only at http://jmb.or.kr.



## Figures and Tables

**Table 1:** Primer sequences used for qRT-PCR.