# Supplementary material for: Effects of Luffa cylindrica (L.) Roem Extract on Microglial Activation-Mediated Mild Cognitive Impairment via Regulation of CREB Signaling Pathway
Source: J Microbiol Biotechnol. 2025 Sep 26;35:e2506049. doi: 10.4014/jmb.2506.06049 (PMC12535862; doi:10.4014/jmb.2506.06049)
Supplement: Supplementary file 1 [file jmb-35-e2506049-supple.pdf]

Figure. 1B

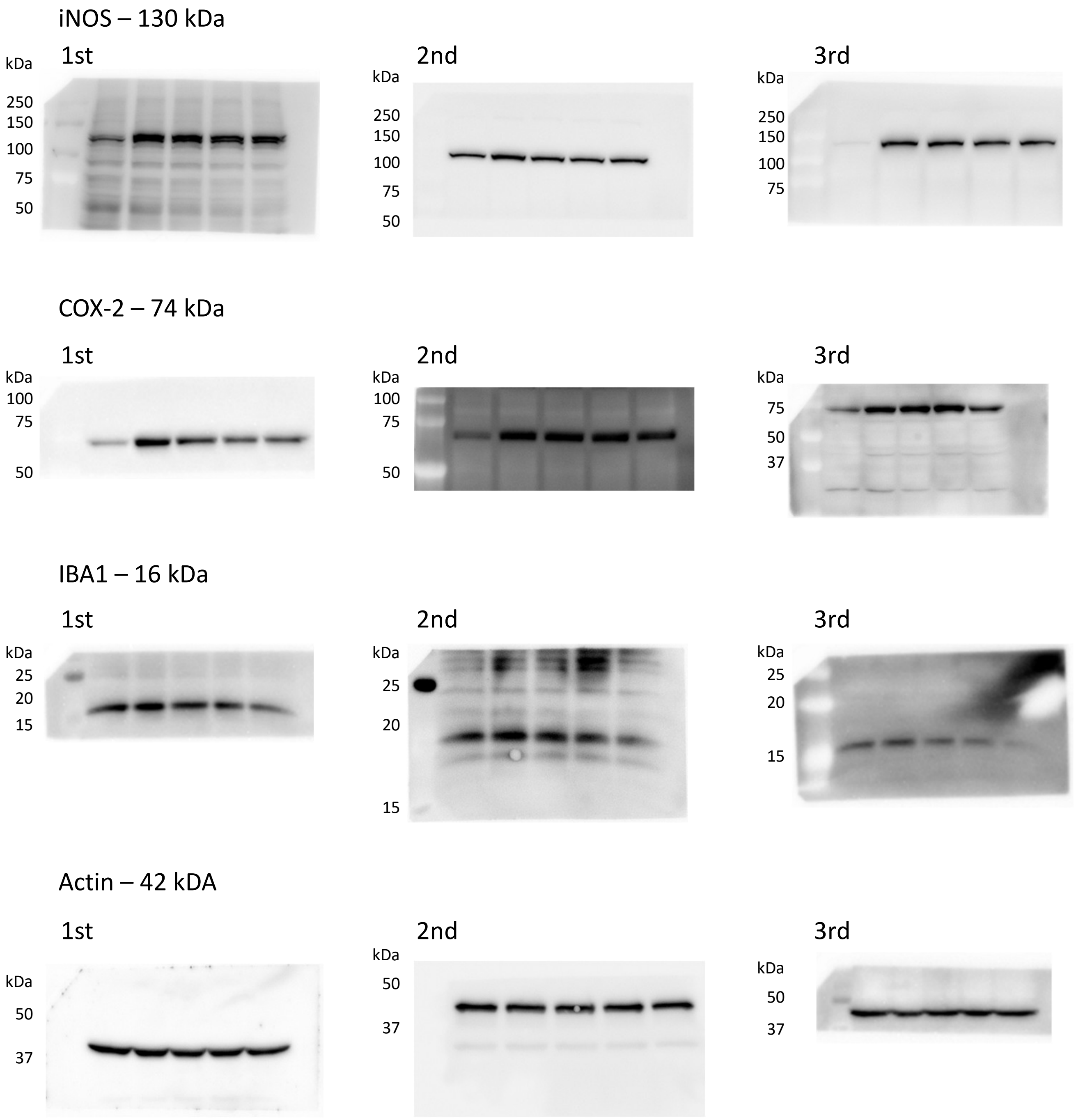

iNOS – 130 kDa
1st
kDa
250
150
100
75
50
2nd
kDa
250
150
100
75
50
3rd
kDa
250
150
100
75
COX-2 – 74 kDa
1st
kDa
100
75
50
2nd
kDa
100
75
50
3rd
kDa
75
50
37
IBA1 – 16 kDa
1st
kDa
25
20
15
2nd
kDa
25
20
15
3rd
kDa
25
20
15
Actin – 42 kDA
1st
kDa
50
37
2nd
kDa
50
37
3rd
kDa
50
37

Figure. 3A

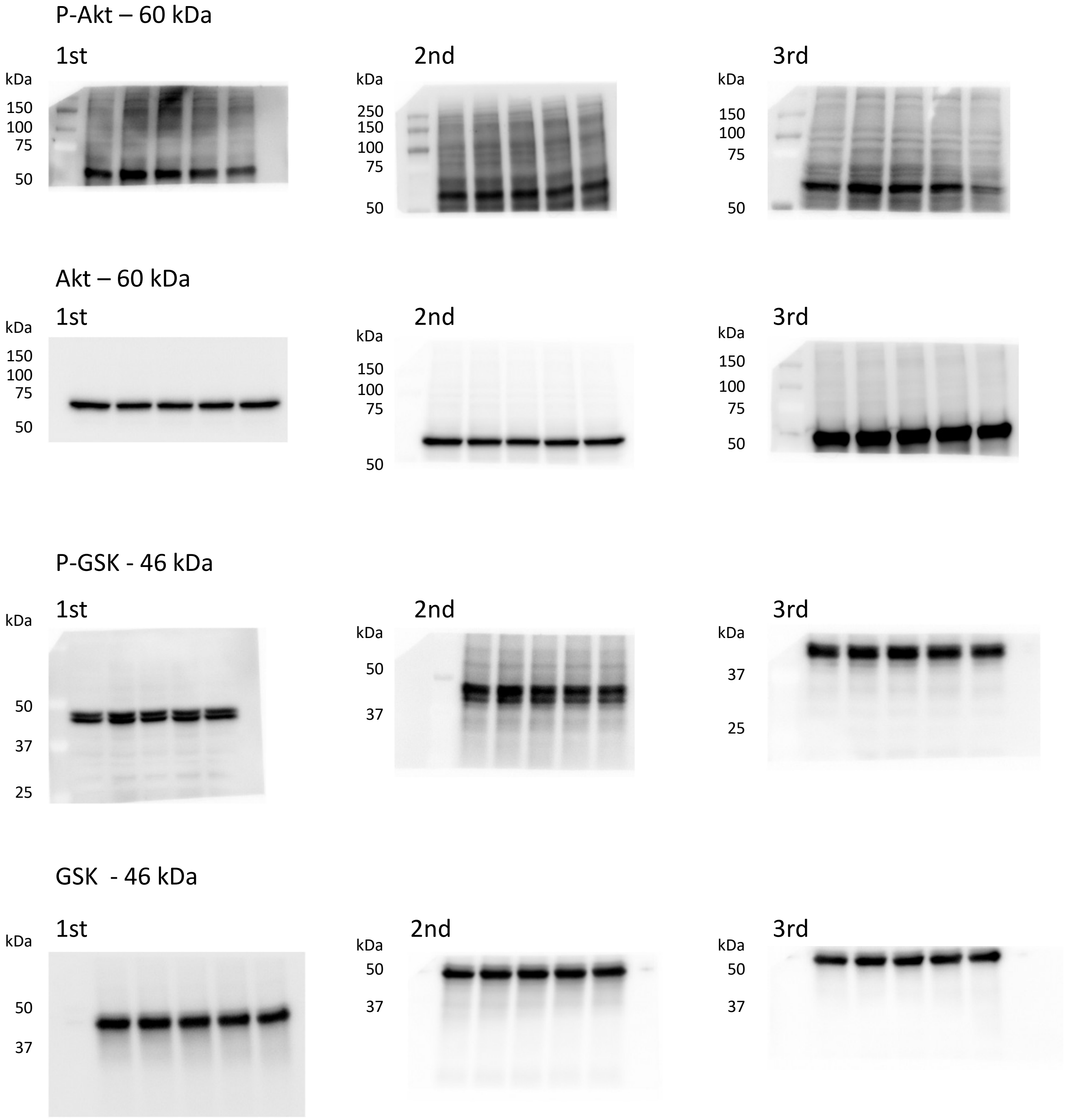

P-Akt – 60 kDa
1st
kDa
150
100
75
50
2nd
kDa
250
150
100
75
50
3rd
kDa
150
100
75
50
Akt – 60 kDa
1st
kDa
150
100
75
50
2nd
kDa
150
100
75
50
3rd
kDa
150
100
75
50
P-GSK - 46 kDa
1st
kDa
50
37
25
2nd
kDa
50
37
3rd
kDa
37
25
GSK - 46 kDa
1st
kDa
50
37
2nd
kDa
50
37
3rd
kDa
50
37

Figure. 3A

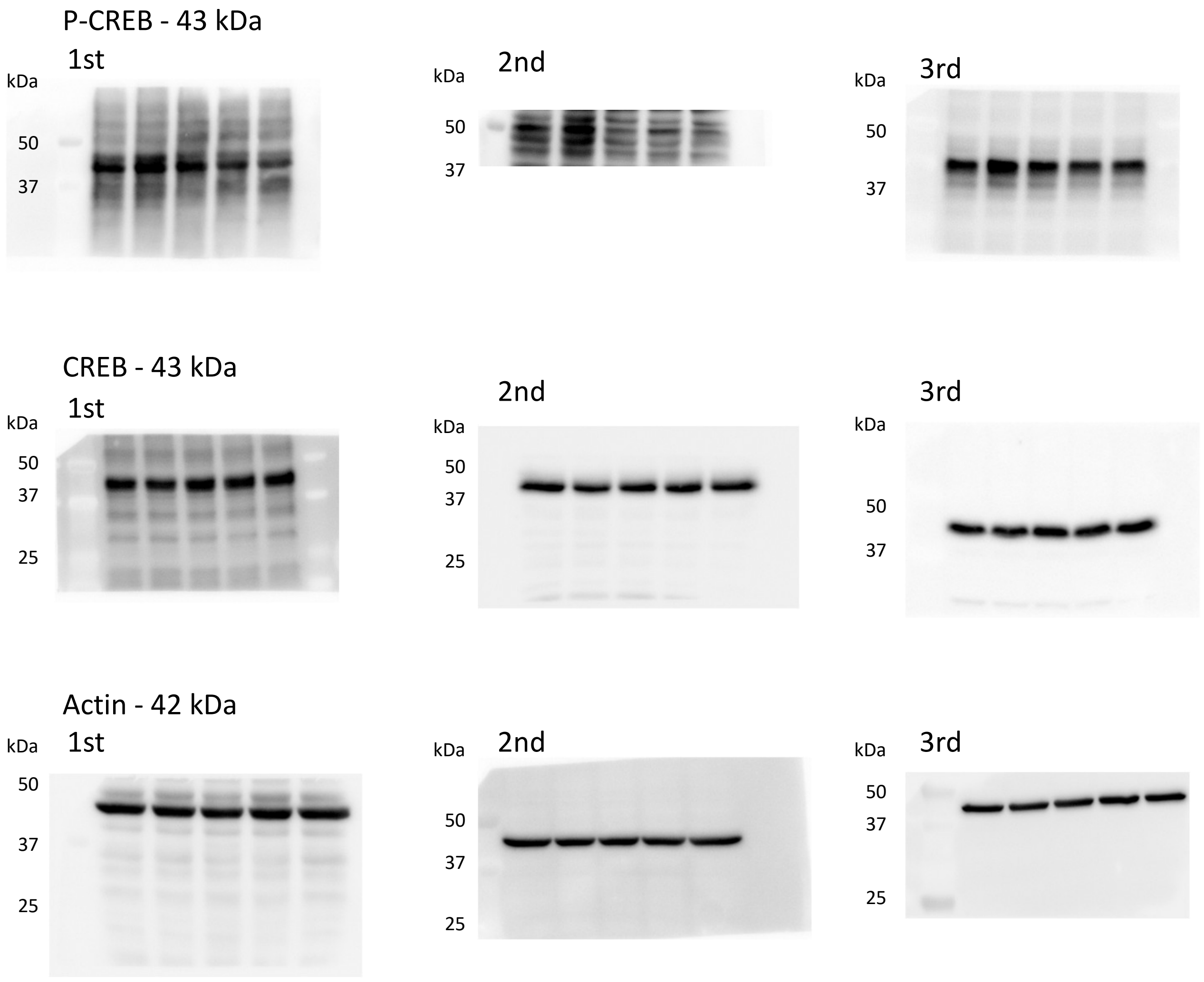
P-CREB - 43 kDa
1st
2nd
3rd
kDa
50
37
CREB - 43 kDa
1st
2nd
3rd
kDa
50
37
25
Actin - 42 kDa
1st
2nd
3rd
kDa
50
37
25

Figure. 3B

iNOS – 130 kDa

1st

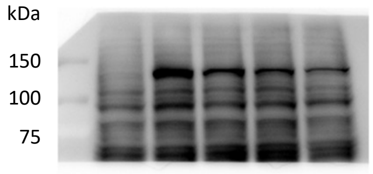

2nd

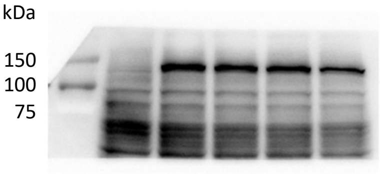

3rd

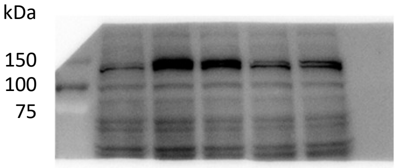

COX-2 – 74 kDa

1st

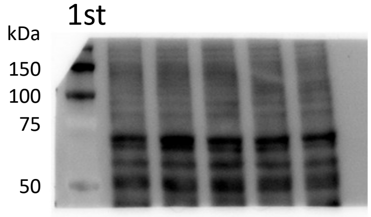

2nd

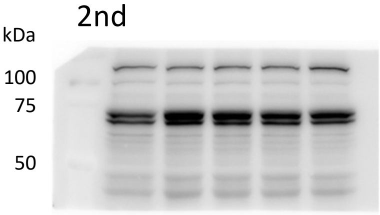

3rd

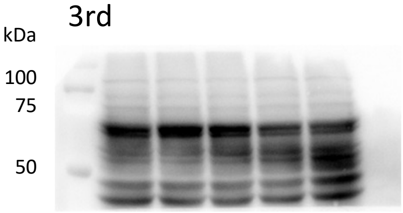

IBA1 – 16 kDa

1st

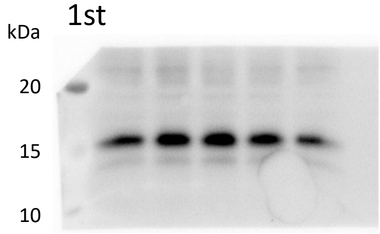

2nd

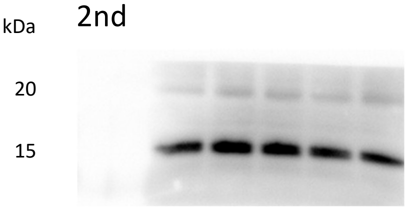

3rd

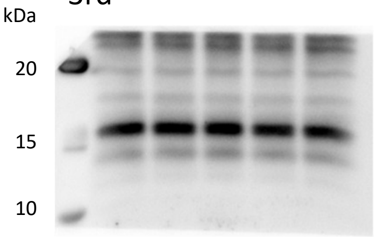

Actin – 42 kDA

1st

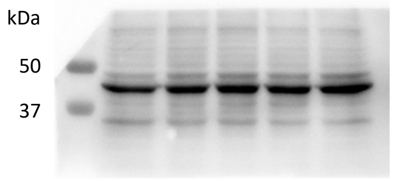

2nd

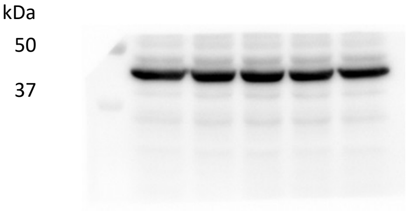

3rd

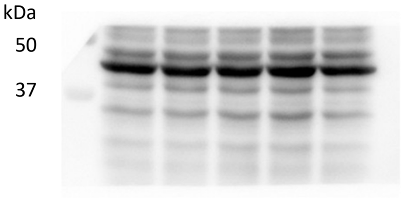

Figure. 4B

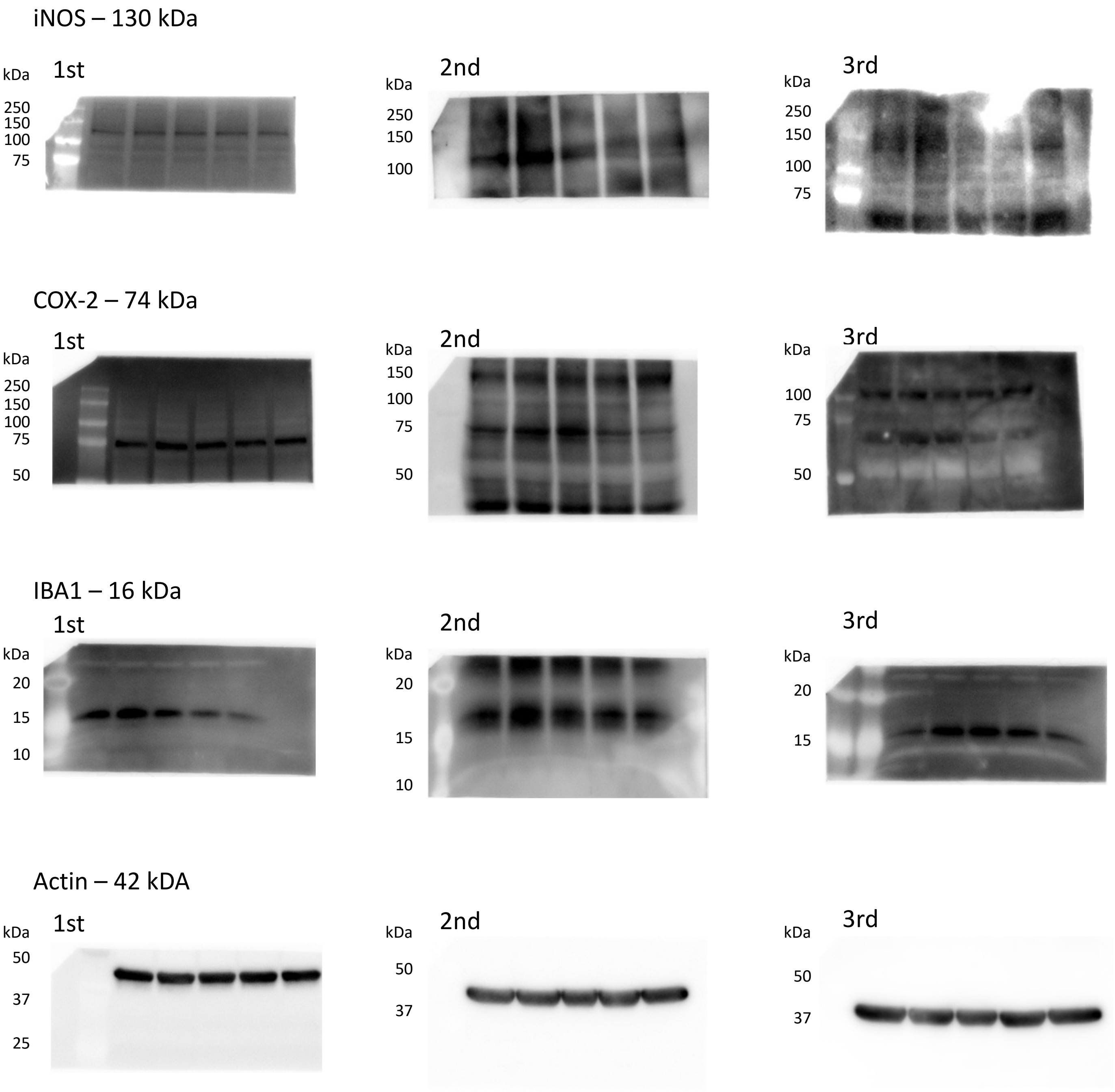
iNOS – 130 kDa
1st
2nd
3rd
kDa
250
150
100
75
COX-2 – 74 kDa
50
IBA1 – 16 kDa
20
15
10
Actin – 42 kDA
37
25

Figure. 5C

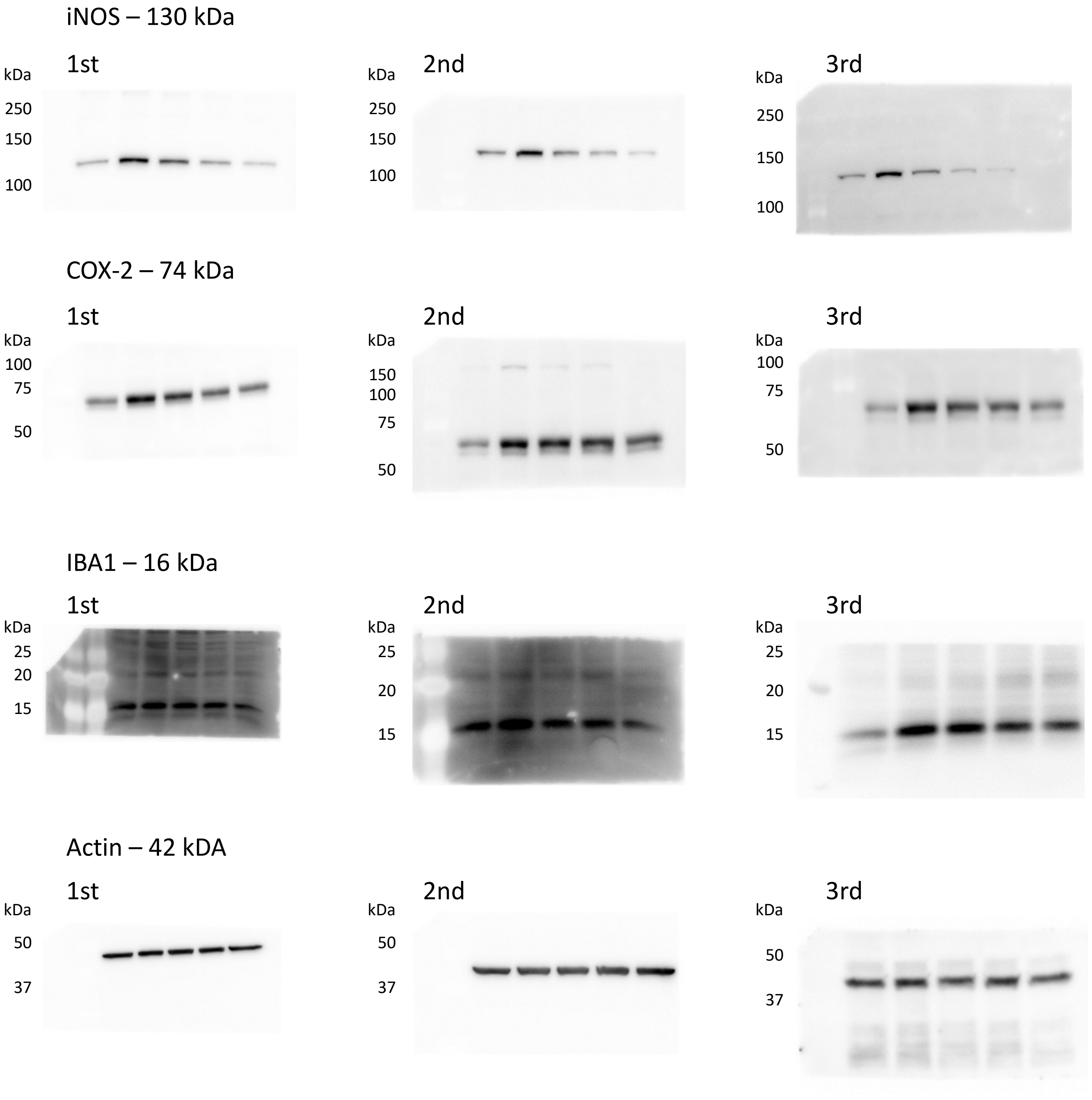
iNOS – 130 kDa
1st
2nd
3rd
kDa
250
150
100
COX-2 – 74 kDa
1st
2nd
3rd
kDa
100
75
50
150
IBA1 – 16 kDa
1st
2nd
3rd
kDa
25
20
15
Actin – 42 kDA
1st
2nd
3rd
kDa
50
37
